# Novel low-kVp beamlet system for choroidal melanoma

**DOI:** 10.1186/1748-717X-1-36

**Published:** 2006-09-11

**Authors:** Carlos Esquivel, Clifton D Fuller, Robert G Waggener, Adrian Wong, Martin Meltz, Melissa Blough, Tony Y Eng, Charles R Thomas

**Affiliations:** 1 Cancer Therapy and Research Center, San Antonio, TX, USA; 2 Graduate Division of Radiological Sciences, Department of Radiology, University of Texas Health Science Center at San Antonio, San Antonio, TX, USA; 3 Department of Radiation Oncology, University of Texas Health Science Center at San Antonio, San Antonio, TX, USA; 4 Department of Diagnostic and Interventional Imaging, University of Texas Health Science at Houston, Houston, TX, USA; 5 Department of Radiation Medicine, Oregon Health & Science University, Portland, OR, USA

## Abstract

**Background:**

Treatment of choroidal melanoma with radiation often involves placement of customized brachytherapy eye-plaques. However, the dosimetric properties inherent in source-based radiotherapy preclude facile dose optimization to critical ocular structures. Consequently, we have constructed a novel system for utilizing small beam low-energy radiation delivery, the Beamlet Low-kVp X-ray, or "BLOKX" system. This technique relies on an isocentric rotational approach to deliver dose to target volumes within the eye, while potentially sparing normal structures.

**Methods:**

Monte Carlo N-Particle (MCNP) transport code version 5.0(14) was used to simulate photon interaction with normal and tumor tissues within modeled right eye phantoms. Five modeled dome-shaped tumors with a diameter and apical height of 8 mm and 6 mm, respectively, were simulated distinct positions with respect to the macula iteratively. A single fixed 9 × 9 mm^2 ^beamlet, and a comparison COMS protocol plaque containing eight I-125 seeds (apparent activity of 8 mCi) placed on the scleral surface of the eye adjacent to the tumor, were utilized to determine dosimetric parameters at tumor and adjacent tissues. After MCNP simulation, comparison of dose distribution at each of the 5 tumor positions for each modality (BLOKX vs. eye-plaque) was performed.

**Results:**

Tumor-base doses ranged from 87.1–102.8 Gy for the BLOKX procedure, and from 335.3–338.6 Gy for the eye-plaque procedure. A reduction of dose of at least 69% to tumor base was noted when using the BLOKX. The BLOKX technique showed a significant reduction of dose, 89.8%, to the macula compared to the episcleral plaque. A minimum 71.0 % decrease in dose to the optic nerve occurred when the BLOKX was used.

**Conclusion:**

The BLOKX technique allows more favorable dose distribution in comparison to standard COMS brachytherapy, as simulated using a Monte Carlo iterative mathematical modeling. Future series to determine clinical utility of such an approach are warranted.

## Background

Choroidal melanoma is the most common intraocular malignancy in adults, originating within the pigmented cells of the choroid [1]. Management of patients with this neoplasm is complex and remains the subject of much discussion. The treatment modality of choice is predicated in part by the size and location of the tumor [2,3].

Until the 1980's, the standard treatment of choroidal melanoma was the removal of the eye by enucleation [4]. Alternative therapies have since been developed to preserve the eye and vision, such as laser photocoagulation, cryotherapy, local resection and radiation therapy. In 1986, the Collaborative Ocular Melanoma Study (COMS) was initiated to address the role of radiotherapy versus enucleation[5]. This fifteen-year long study examined patients with choroidal melanoma, treated with a brachytherapy procedure that uses gold episcleral plaques containing Iodine 125 (I-125) seeds for the treatment of small- to medium-sized tumors. In a surgical procedure, an eye-plaque is placed on the scleral surface of the eye adjacent to the tumor, and left in place for three to seven days to deliver a therapeutic dose of radiation. The plaque size and the number of seeds utilized depend on the size and location of the tumor in the eye to be irradiated. The American Brachytherapy Society (ABS) recommends a minimum tumor dose of 85Gy to the apex of the tumor [6]. The plaque treatment is often successful in controlling the tumor[2,7,8]. However, sequelae are often seen post-therapy; after approximately three years, the patient may lose functional vision in the eye, due to radiation damage near the optic nerve and/or the region of the fovea resulting from direct irradiation by the I-125 seeds [9-12]. This radiation-induced toxicity is an unavoidable by-product of the present plaque system. Enucleation still remains a standard treatment for large choroidal melanomas or in cases where radiation therapy fails[13].

This study proposed the use of a small collimated beam of low energy x-rays, comparable to the average emitted energy by I-125, for the treatment of choroidal melanoma[14]. Our system, the Beamlet Low-kVp X-ray, or "BLOKX" system, offers potential dose distributions amenable to tumor control, ideally with preservation of vision and the eye itself. The BLOKX uses one or several small beams to minimize primary irradiation to normal structures in the eye. In addition, this procedure will not require hospitalization. The BLOKX can direct a small beam of x-rays at a simulated eye tumor, while at the same time sparing critical structures of the eye. Primary beams from the BLOKX may be targeted such that the optic nerve region is spared, with only scattered radiation reaching critical intraocular structures.

As part of a program to optimize the technical specifications of the BLOKX system, we sought to characterize potential dosimetric advantages available using this technique. In general, Monte Carlo transport techniques are recognized in radiotherapy as valuable method for extrapolation of computed patient dose. Monte Carlo iterative analysis aids in improving the accuracy of clinical dosimetry by providing more realistic data through modeling of multiple complex parameters. Consequently, we have sought to determine whether, based on Monte Carlo modeling, dosimetric profile enhancement may be realized in using the BLOKX system in comparison to an established clinical standard, episcleral eye-plaque brachytherapy, in a simulation scenario designed to approximate clinically relevant parameters for a stereotypical choroidal melanoma.

Specific aims included

1. Evaluation of BLOKX, a modeled novel system with the mechanical ability to move in three-dimensional space, such that a small beam of x-rays can be directed at an eye tumor while sparing the optic nerve region

2. Comparison of dosimetric models derived from Monte Carlo simulations using the BLOKX to doses using COMS eye-plaque technique.

## Methods

### BLOKX system

**Figure 1:**
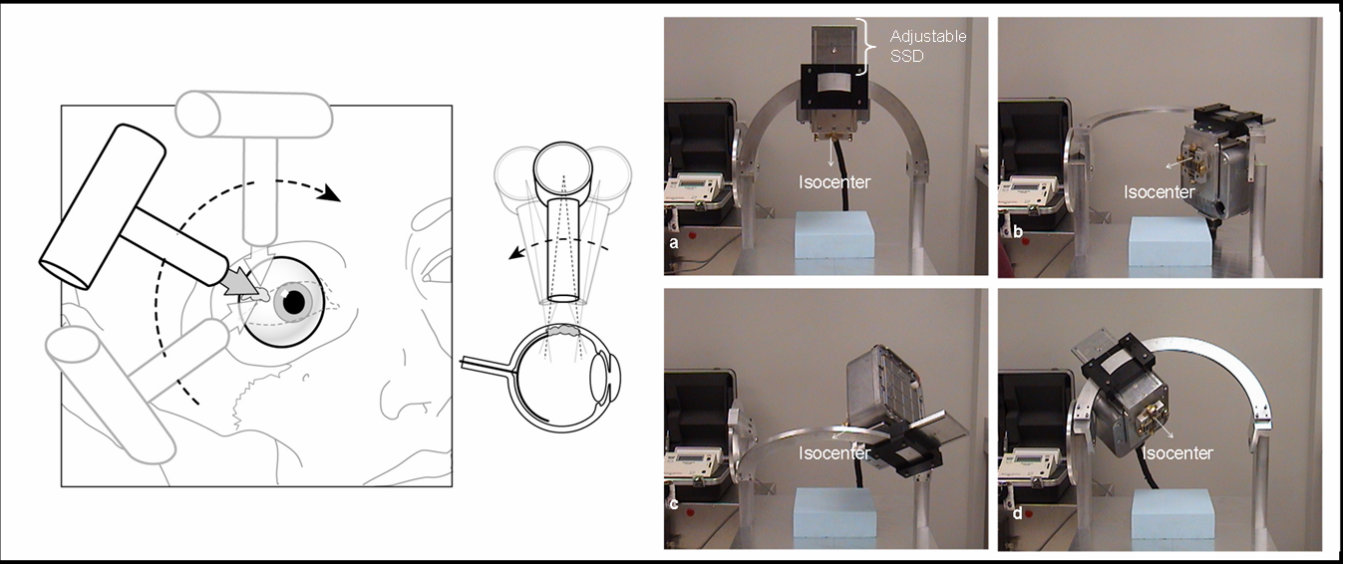
Diagrammatic representation of BLOKX rotational technique (left); Siemens Orthopantomograph x-ray unit model #0P 10 A reconstructed with the mechanical ability to move in three-dimensional space about an isocentric point (right). The source to axis distance is adjustable, with a 14 cm range.

The BLOKX was created from a pre-existing Siemens Orthopantomograph (Model #0P 10 A) unit, reconstructed with the mechanical ability to move in three-dimensional space about an isocentric point. Figure 1 shows a picture of the BLOKX device. The system operates in the 60–90 kVp range with tube currents between 5 to 12 mA, and beam quality may be modified by 2.8 mm aluminum filtration. Through careful geometric planning, a desirable position may be chosen to deliver maximum dose to the tumor, while limiting exposure to other critical non-target ocular structures. The BLOKX unit's output was measured with a Radcal control unit (Model Number 9010) with an ionization chamber/electrometer (Model Number 9060) for different kVp and mA settings. Reproducibility, accuracy, and the half-value layer were measured for each kVp and mA setting available on the unit. After evaluation, the BLOKX was operated at 75 kVp, 12 mA, and a half-value layer of 2.8 mm of aluminum added filtration, chosen as the settings that would provide an effective energy similar to that of I-125.

### Monte Carlo code & simulation

For this study, the Monte Carlo N-Particle (MCNP) transport code version 5.0[15] was used (X-5 Monte Carlo Team, Los Alamos, NM). The code was obtained from the Oak Ridge National Laboratory through the Radiation Safety Information Computational Center[15]. The code has generalized 3-D geometry capabilities using first- and second-degree surfaces and forth-degree elliptical tori, with extensive cross section libraries. MCNP5 has two models for photon interaction: simple and detailed. The detailed physics treatment includes coherent scattering and fluorescent photons after photoelectric absorption, while the simple treatment ignores both. The detailed model is always used as the default model for photons with energies less than 100 MeV.

The MCNP5 program was run on a Dell model DHM GX260 Optiplex Intel ^® ^Pentium 4 personal computer system running at 2.4 GHz, with a 40 GB hard drive, and supported by Windows PC with a Lahey compiler. Visual Editor[16] (Visual Editor Consultants, Richland, WA) and Sabrina[15] (White Rock Science, Los Alamos, NM) software were used to image the setups. Several MCNP input files were written for the aforementioned treatment system and tumor locations in the eye. The input data included the dimensions and location of the tumor, composition and location of the eye structures and the source/plaque description. Dose rates were determined for critical intraocular structures such as the lens, macula, optic disc, base and apex of the tumor. Each MCNP file was benchmarked by COMS-ROCS treatment planning calculations or measurements.

**Figure 2:**
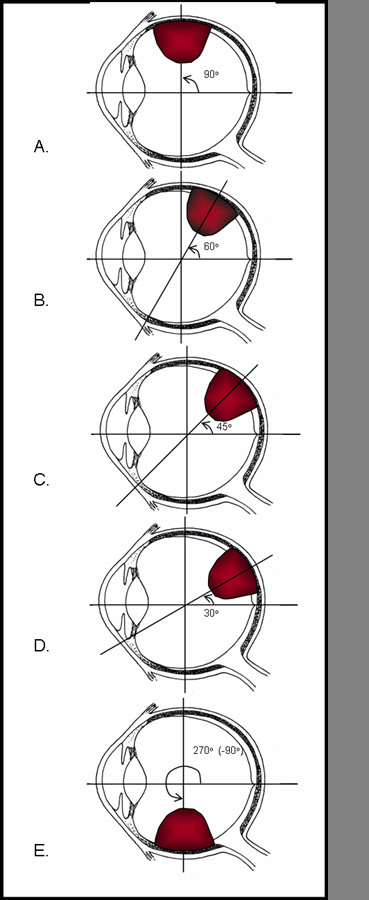
Spatial location of intra-ocular tumor models, showing 90°(A), 60°(B), 45°(C), 30°(D) and 270°(E) positions.

Five MCNP input files were written to model the right eye, with a tumor placed at five different positions with respect to the macula (Figure 2). The modeled eye had inner and outer diameters of 11 and 12 mm, respectively. The parts of the eye that were modeled included the sclera, macula, optic nerve, lens, cornea, aqueous humor and vitreous humor. The whole eye was considered to be made of water equivalent tissue. A dome-shaped tumor with a diameter and apical height of 8 mm and 6 mm, respectively, was modeled.

The selected model tumor was a medium-size dome shaped lesion representative of a stereotypical choroidal melanoma. The basal diameter and apical height of this tumor were 8 mm and 6 mm, respectively. Five stereotypical tumor locations within the eye were used for the project: 30, 45, 60, 90 and 270 degrees from the macula. Doses to critical structures were calculated for the sclera at the base of the tumor, the prescription point at the apex of the tumor, the macula, the optic nerve, and the center of the lens. Critical structures and the center of the dome-shaped tumor were all located in the same axial plane. For this study, all the calculation points for the tumor and critical structures were localized in the X-Y plane, with the inner sclera surface at the center of the tumor base is the origin of a Cartesian (x, y, z) coordinate system, (0, 0, 0) mm. The coordinates for tumor apex were (6, 0, 0) mm. The coordinates for the macula, optic nerve and the lens varied consonant with tumor location. In this study, we used a dome-shaped tumor with a circular base, so that the tumor base dimensions in both the macula direction and the optic disc direction were equivalent in each tumor position. The MCNP *f8 tally was used to determine the energy deposited in five spherical tally cells with 0.5 mm radius. These tally cells were placed at the tumor base, tumor apex, lens, macula, and at the center of the optic disc.

### A. BLOKX model parameters

**Figure 3:**
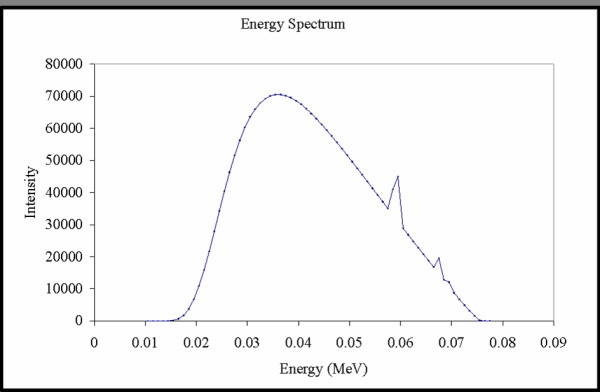
Experimentally derived energy spectrum of BLOKX utilized for Monte Carlo calculations.

The BLOKX was operated at 75 kVp, 12 mA, and a half-value layer of 2.8 mm of aluminum added filtration. These parameters were utilized to characterize comparable inputs for Monte Carlo simulations. A single fixed field size, 9 × 9 mm^2^, was chosen to encompass the whole tumor with a 1 mm margin of error. Figure 3 shows the energy spectrum of the x-ray beam modeled in the MCNP setup.

**Figure 4:**
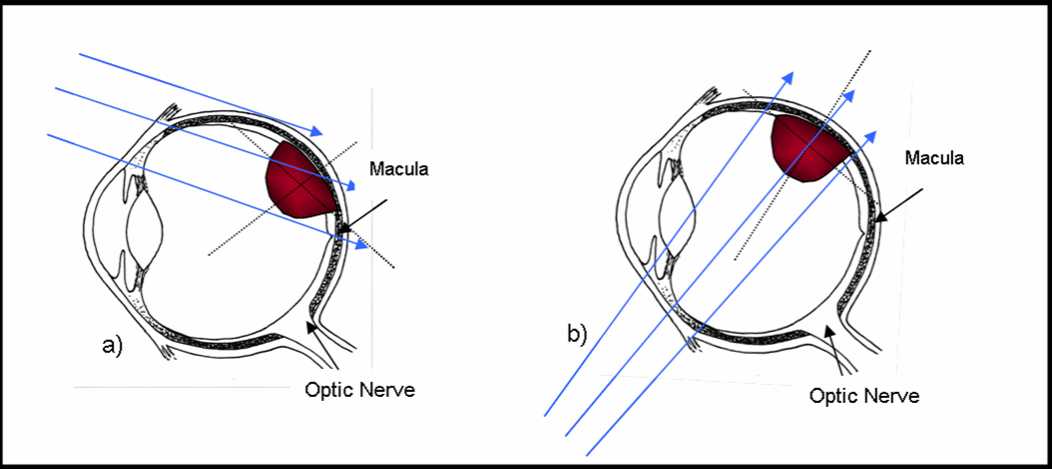
Axial view beam directions for tumor at 45° position; a) Original x-ray beam setup for irradiating the tumor based on TLD measurements, b) Revised x-ray beam direction used to spare direct irradiation of the macula.

Five MCNP input files were written to model absorbed dose measurements made in a right eye phantom irradiated by BLOKX. Each input file modeled a one of the five tumor locations. Two beam directions were investigated in MCNP for the tumor located at 45 degrees with respect to the macula, presented in Figure 4.

The center of the tumor was simulated at isocenter. The x-ray source was modeled at 20 cm from isocenter. The setup is presented in Figure 1. In addition, an MCNP file was written to model the x-ray's percent depth dose in a plastic eye phantom for a superficial depth of up to 2 cm.

The MCNP *f8 energy deposition tally was used to score the dose rate in five spherical tally cells with a 0.5 mm radius. These five tally cells were located at the base of the tumor, the tumor's apex, at the center of the optic nerve, in the middle of the lens and at the fovea (or macula). Iterative simulation was permitted to run for approximately 24 hours for each setup to assure that the tally passed the requisite ten statistical checks.

### B. Absorbed dose determination using episcleral plaque brachytherapy

A 12 mm gold-alloy plaque was modeled as being placed on the scleral surface of the eye adjacent to the tumor. The composition and dimensions of the plaque were determined according to COMS protocol. Each plaque contained eight radioactive I-125 seeds with the same apparent activity of 8 mCi. Each I-125 seed was treated like a point-source. The seeds arrangement is similar to those used in the COMS protocol. All five input files used the same effective energy of 0.0274 MeV and emission yield of 1.523 for the radiation source.

Five MCNP input files were written to model absorbed dose measurements made using a gold-manufactured eye-plaque and plastic eye phantom with the tumor placed at different locations in the eye. The files ran for 30 hours for each tumor location to assure that the tally passed all ten requisite statistical checks.

## Results

### Absorbed dose determinations using the Monte Carlo simulation of the eye plaque procedure

Doses to the sclera at the base of the tumor ranged from 495.3 to 505.6 Gy and were nearly six times greater than the dose to the tumor's apex. The optic nerve doses ranged from 11.8 to 52.2 Gy as the tumor's location was progressively moved closer to the optic nerve. The dose to the macula also increased from 16.7 to 204.7 Gy, as the tumor's location moved to the rear of the eye. The dose to the lens decreased from 23.6 to 12.0 Gy as the tumor was moved more posterior. Uncertainties in dose measurements were kept under 6%. Uncertainties are dependent upon the number of photons depositing dose in the dose bin detectors used in the simulation. As the dose bin detectors are located further away from the source, the uncertainty of measurement increases.

### MCNP simulation of the BLOKX

**Figure 5:**
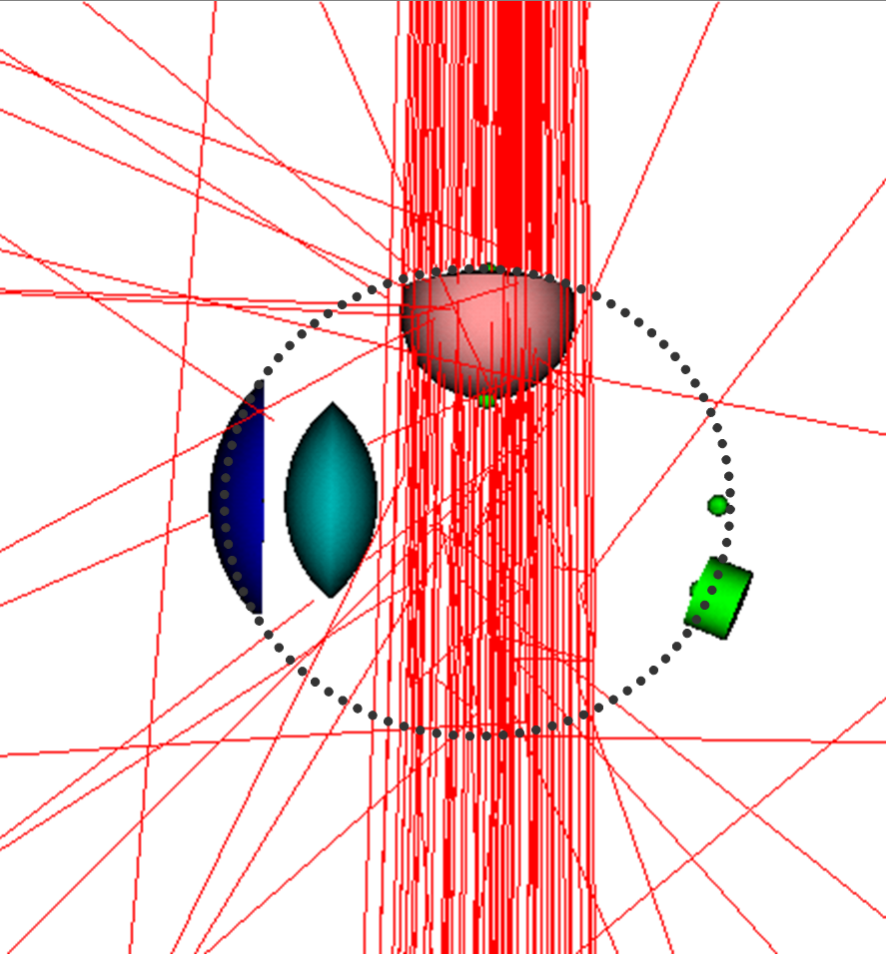
MCNP output simulation of BLOKX procedure.

Doses to the tumor base, apex of the tumor, macula, optic nerve and center of the lens can be seen in Table 1. The doses to the base of the tumor were 87.1, 101.8 and 102.8 Gy respectively, for tumors located at 60, 90 and 270° with respect to the macula. For tumors located at 45 and 30° with respect to the macula, minimum dose to the base of the tumor and all doses to other structures were normalized to the base of the tumor which received 85 Gy. The apex of the tumor received between 93.1 to 112.0 Gy for these locations. The macula received doses between 1.5 to 3.2 Gy for tumors located at 60, 90, and 270° with respect to the macula. For the 45 and 30° tumor locations, the macula received 51.0 and 70.4 Gy respectively. Another simulation was run for the tumor located at 45° from the macula. This time the BLOKX was repositioned and the beam was aimed at the tumor from another direction (Figure 4). A drop in the dose to the macula, from 51.0 to 4.0 Gy was observed. The optic nerve received doses between 1.2 to 2.9 Gy and was well below the recommended dose limit of 10 Gy for all tumor locations. The MCNP-derived doses to the lens range from 1.8 to 16.54 Gy. The uncertainties affecting the measurements were estimated to be approximately 0.32 to 3.1%. Figure 5 illustrates the MCNP simulations of the BLOKX irradiating the tumor in the eye. The primary beam is directed at the tumor. Only the scattered radiation will reach critical structures in the eye.

**Table 1:** MCNP derived results for BLOKX model.

	Location	Dose (Gy)	Uncertainty (%)
Tumor 90° from macula	Base	101.8	0.32
	Apex	85.0	0.35
	Macula	1.5	2.64
	Optic Nerve	1.6	2.54
	Lens	3.3	1.75

Tumor 60° from macula	Base	87.1	0.36
	Apex	85.0	0.36
	Macula	3.1	1.9
	Optic Nerve	1.2	3.06
	Lens	2.4	2.19

Tumor 45° from macula	Base	85.0	0.4
	Apex	93.1	0.38
	Macula	51.0	0.52
	Optic Nerve	1.8	2.72
	Lens	3.6	1.9

Tumor 45°R* from macula	Base	85.0	0.46
	Apex	112.0	0.4
	Macula	4.0	2.1
	Optic Nerve	2.9	2.45
	Lens	7.1	1.57

Tumor 30° from macula	Base	85.0	0.42
	Apex	99.1	0.39
	Macula	70.4	0.46
	Optic Nerve	3.0	2.23
	Lens	4.7	1.75

Tumor 270° from macula	Base	102.8	0.31
	Apex	85.0	0.35
	Macula	3.2	1.8
	Optic Nerve	2.9	1.88
	Lens	1.8	2.36

### MCNP simulation of manufactured eye-plaque

**Figure 6:**
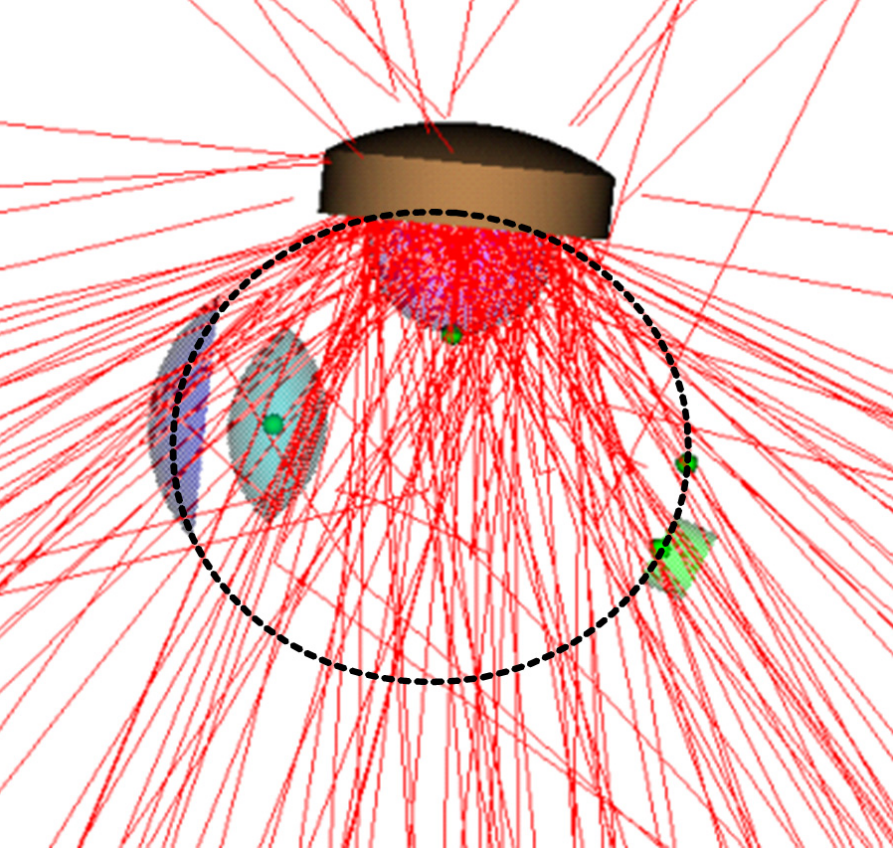
MCNP output simulation of eye-plaque procedure.

Dose calculations to the tumor base, apex of the tumor, macula, center of the optic disc, and the center of the lens derived from the Monte Carlo simulation are shown in Table 2. Doses of 85 Gy were prescribed to the tumor's apex. Doses to the tumor base at the sclera ranged from 335.5 to 338.6 Gy and are nearly five times greater than those at the apex. As the tumor moved closer to the optic nerve and macula, the doses to these locations increased. MCNP shows that the dose increased from 5.5 to 16.9 Gy for the optic nerve and 11.0 to 152.9 Gy for the macula. The modeled eye-plaque delivered nearly double the prescription dose to the macula when the tumor was located 30° from the macula. The lens dose decreased from 11.0 to 5.6 Gy as the tumor was moved to the posterior part of the eye. Uncertainty of dose calculations were all under 7% and decreased as the dose bin detectors were located closer to the source. An illustration of the MCNP simulation of the eye-plaque procedure can be seen in Figure 6. Both primary and scattered radiation will reach critical structures in the eye.

**Table 2:** MCNP derived results for simulated eye-plaque model.

	Location	Dose (Gy)	Uncertainty (%)
Tumor 90° from macula	Base	338.6	0.89
	Apex	85.0	1.78
	Macula	11.0	5.00
	Optic Nerve	5.5	6.97
	Lens	11.0	4.59

Tumor 60° from macula	Base	338.2	0.89
	Apex	85.0	1.77
	Macula	20.2	3.65
	Optic Nerve	11.1	4.96
	Lens	7.9	5.80

Tumor 45° from macula	Base	335.3	0.89
	Apex	85.0	1.77
	Macula	39.3	2.61
	Optic Nerve	15.7	4.18
	Lens	6.8	6.22

Tumor 30° from macula	Base	335.5	0.89
	Apex	85.0	1.77
	Macula	152.9	1.3
	Optic Nerve	16.9	3.98
	Lens	5.6	6.82

Tumor 270° from macula	Base	338.1	0.89
	Apex	85.0	1.78
	Macula	11.0	4.99
	Optic Nerve	14.7	4.33
	Lens	12.8	4.59

### MCNP X-ray calculations comparison with MCNP eye-plaque calculations

**Figure 7:**
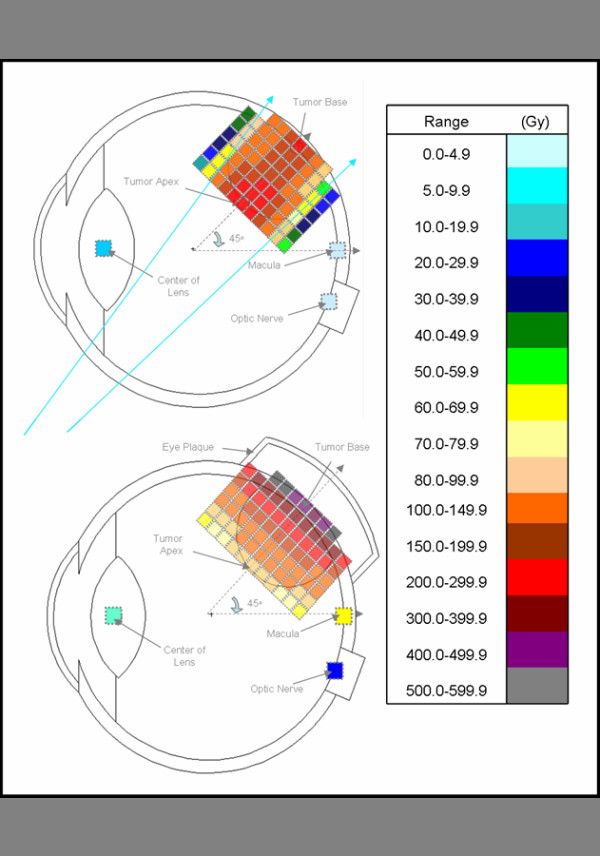
Tumor volume dose distribution comparison of BLOKX and eye plaque methods for tumor at 45° position, with legend.

**Figure 8:**
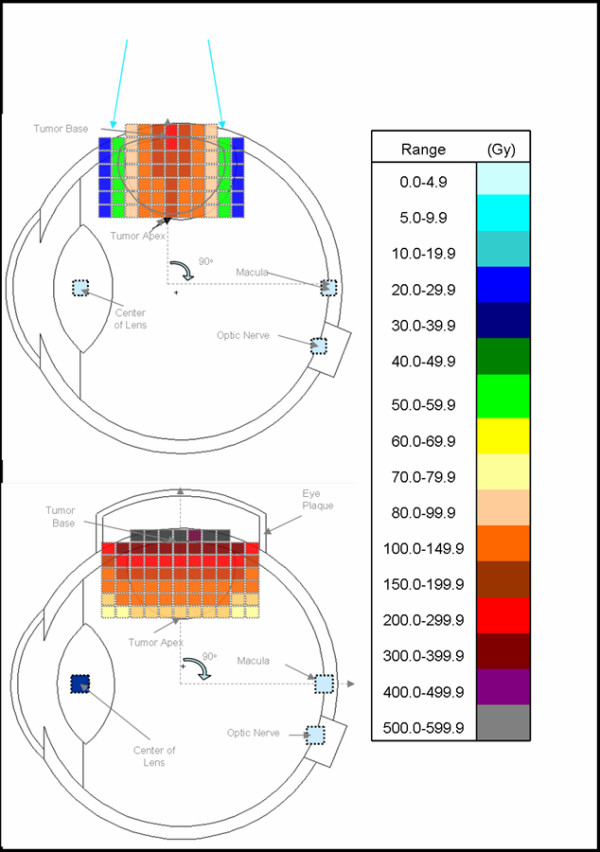
Tumor volume dose distribution comparison of BLOKX and eye plaque methods for tumor at 90° position, with legend.

A comparison of the MCNP-derived doses for the BLOKX and the eye-plaque procedure is summarized in Table 3. For the eye-plaque procedure, all doses to the apex were normalized to the prescribed dose of 85 Gy. The MCNP-derived doses to the base of the tumor ranged slightly, from 335.3 to 338.6 Gy, for the eye-plaque procedure. For the BLOKX, the minimum prescribed dose of 85 Gy was delivered to the point that received the lowest dose rate. At the 30 and 45° tumor location, the base of the tumor received 85 Gy and the apex received 99.1 and 93.1 Gy. The tumor's apex received 85 Gy for all other tumor locations and the tumor base doses ranged from 87.1 to 102.8 Gy for the x-ray procedure. A reduction of dose of at least 69% was seen when using the x-ray procedure when compared to the eye-plaque procedure. Due to the modest dose gradient of the x-ray beam, a more uniform dose distribution can be delivered to the tumor. For illustrative purposes, diagrammatic comparisons between techniques for the 45° and 90° tumor positions are illustrated in Figures 7 and 8.

**Table 3:** Comparison of MCNP calculations by treatment method.

	MCNP Eye-plaque		MCNP BLOKX		
		
	Location	Dose (Gy)	Location	Dose (Gy)	Difference (%) *
Tumor 90° from macula	Base	338.6	Base	101.8	69.9
	Apex	85.0	Apex	85.0	0.0
	Macula	11.0	Macula	1.5	86.0
	Optic Nerve	5.5	Optic Nerve	1.6	71.0
	Lens	11.0	Lens	3.3	69.7

Tumor 60° from macula	Base	338.2	Base	87.1	74.2
	Apex	85.0	Apex	85.0	0.0
	Macula	20.2	Macula	3.1	84.4
	Optic Nerve	11.1	Optic Nerve	1.2	89.4
	Lens	7.9	Lens	2.4	70.3

Tumor 45° from macula	Base	335.3	Base	85.0	74.7
	Apex	85.0	Apex	93.1	-9.5
	Macula	39.3	Macula	51.0	-29.7
	Optic Nerve	15.7	Optic Nerve	1.8	88.4
	Lens	6.8	Lens	3.6	46.7

Tumor 45°R* from macula	Base	335.3	Base	85.0	74.7
	Apex	85.0	Apex	112.0	-31.7
	Macula	39.3	Macula	4.0	89.8
	Optic Nerve	15.7	Optic Nerve	2.9	81.7
	Lens	6.8	Lens	7.1	-4.0

Tumor 30° from macula	Base	335.5	Base	85.0	74.7
	Apex	85.0	Apex	99.1	-16.6
	Macula	152.9	Macula	70.4	53.9
	Optic Nerve	16.9	Optic Nerve	3.0	82.4
	Lens	5.6	Lens	4.7	16.9

Tumor 270° from macula	Base	338.1	Base	102.8	69.6
	Apex	85.0	Apex	85.0	0.0
	Macula	11.0	Macula	3.2	71.2
	Optic Nerve	14.7	Optic Nerve	2.9	80.4
	Lens	12.8	Lens	1.8	85.8

For the eye-plaque procedure, the MCNP-derived doses for the macula increased from 11.0 to 152.9 Gy as the tumor-model was moved to the rear of the eye. The MCNP-derived doses for the macula, when using the BLOKX, at 90, 60, 45, 30 and 270° tumor locations were 1.5, 3.1, 51.0, 70.4 and 3.2 Gy respectively. For the 30° tumor location, a 53.9% reduction of dose is seen when using the BLOKX. However, the dose to the macula was still quite high, 51.0 Gy. At the 45° tumor location, the dose to the macula from the BLOKX was greater than the dose given by the eye-plaque. In the original setup, the macula is irradiated by some of the primary x-ray beam. A revised MCNP simulation was created for BLOKX for the 45° tumor, demonstrating a capacity for dose reduction vs. the eye-plaque method. The revised MCNP simulation shows a significant reduction of dose, 89.8%, to the macula (4.0 Gy) when compared to the eye-plaque procedure (39.3 Gy).

Derived doses to the optic nerve using the eye-plaque procedure increased from 5.5 to 16.9 Gy as the tumor model was moved to the posterior part of the eye. The BLOKX derived doses ranged from 1.2 to 3.0 Gy. A minimum 71.0 % decrease in dose to the optic nerve occurred when the BLOKX was used.

MCNP-derived doses to the lens using the eye-plaque procedure generally decreased from 12.8 to 5.6 Gy as the tumor model location was moved toward the eye's posterior. The BLOKX's derived doses to the lens also showed a general decrease in dose from 7.1 to 1.8 Gy.

## Conclusion

The success of treating choroidal melanoma depends on survival, vision, and the quality of life. The Collaborative Ocular Melanoma Study has shown that the eye-plaque method is both an eye-sparing and vision-sparing technique for the diagnosed patient[5]. However, within three years, the treated patient may lose functional vision in the eye because of radiation damage near the optic nerve and/or the region of the fovea[3,17,18], with minimal gains seen in overall mortality[19]. Consequently, methodologies which provide dosimetric superiority are viable areas of exploration, with potential for technical circumvention of morbidity with equivalent local control and mortality[19,20].

The low-kVp x-ray system has the ability to move in three-dimensions and direct a small conformal beam of x-rays to the tumor thereby directly irradiating the tumor and sparing normal tissues. Further examinations can be made using Monte Carlo simulations. Preliminary work has shown that by modifying or changing the x-ray field size can further reduce the dose to critical structures such as the optic nerve, macula, and the lens when compared not only to the COMS eye-plaque procedure but to those measured and calculated by the BLOKX procedure that was investigated in this research.

In our dataset the BLOKX system demonstrates a significant diminution of dose to the optic nerve and the macula. The dose reduction to the optic nerve and the macula may result in retaining vision and/or visual acuity, as doses to the center of the lens were kept below 10 Gy. In some cases, there was a further reduction of dose to the center of the lens. Doses to the base of the tumor were also dramatically decreased, with a minimum 70% reduction of dose to the center of the tumor base observed using the BLOKX. This substantial reduction of dose to the tumor base holds the promise to prevent scleral necrosis, maculopathy, or retinopathy via dose reduction to critical points within the eye[10,21].

The BLOKX has the ability to modify the x-ray beam and direct it in many directions to produce a conformal dose to the tumor. The radiation reaching the optic nerve, macula and the lens may be limited to scattered radiation, unlike the COMS method where one or more of the seeds will directly irradiate these critical structures. By having the ability to move the BLOKX about isocenter and having some ability to rotate the eye, the BLOKX can deliver a single x-ray beam of a fixed field size and deliver a minimum prescribed dose to the tumor in a relatively short amount of time for most tumor locations.

The American Brachytherapy Society recommends treating with a minimum of 85 Gy to the apex of the tumor. In order to not exceed tolerances of critical structures, lower doses (70–80 Gy) have been used with successful results [22,23]. For tumors with large apical heights, steep dose gradients have been seen. For episcleral plaque brachytherapy, the average tumor dose is much greater for tumors of large apical height than those with smaller heights. The BLOXK system will deliver average tumor doses that are comparable to episcleral plaques using individual source collimation [24]. Astrahan et al. have show that such plaques produce "a more homogeneous dose distribution in the tumor, reduces scleral dose by up to 50% as compared to conventional designs, and significantly reduces radiation dose to uninvolved structures adjacent to the plaque" without compromising tumor control [24].

Further investigation into examining more tumor locations, using multiple field sizes and beam delivery from different locations for different size tumors may provide interesting and satisfactory justification for clinical utilization for the BLOKX system. Future work can incorporate the use of an x-ray unit with higher current capability (20–30 Amps.) This in turns provides higher x-ray output and will dramatically decrease the treatment time to minutes when compared to the currently used eye-plaque procedure. In addition, computed tomography and a treatment planning system for accurate dosimetry will also be investigated as well as accurate immobilization of the eye during imaging and treatment. Currently, local anesthesia and external immobilization devices used in proton beam therapy of choroidal melanoma have shown promising results in the immobilization of the eye during treatment. This method of radiation therapy represents a practical alternative for the treatment of choroidal melanoma that might eventually be available at many cancer therapy centers, with a modicum of space and capital investment.
